# Natural products that target p53 for cancer therapy

**DOI:** 10.1007/s11418-025-01906-6

**Published:** 2025-04-28

**Authors:** Sachiko Tsukamoto

**Affiliations:** Department of Natural Medicines, Graduate School of Pharmaceutical Sciences, Kumamoto University, 5-1 Oe-Honmachi, Kumamoto, 862-0973 Japan

**Keywords:** Natural products, p53 reactivation, Tumor suppressor, Cancer therapy

## Abstract

Wild-type p53 acts as a tumor suppressor, but p53 is frequently mutated and inactivated in tumor cells, promoting cancer progression, invasion, and metastasis. Thus, compounds that reactivate p53 may be leveraged for cancer treatment, and the development of drugs targeting p53 reactivation is actively progressing. Notably, natural products exhibit diverse structures and biological activities and are used as therapeutic agents for various diseases worldwide. This review discusses the natural products that inhibit p53 degradation through p53–Mdm2 interaction, promote p53 reactivation by inducing conformational changes, and exhibit p53-dependent growth inhibition.

**Graphical abstract:**

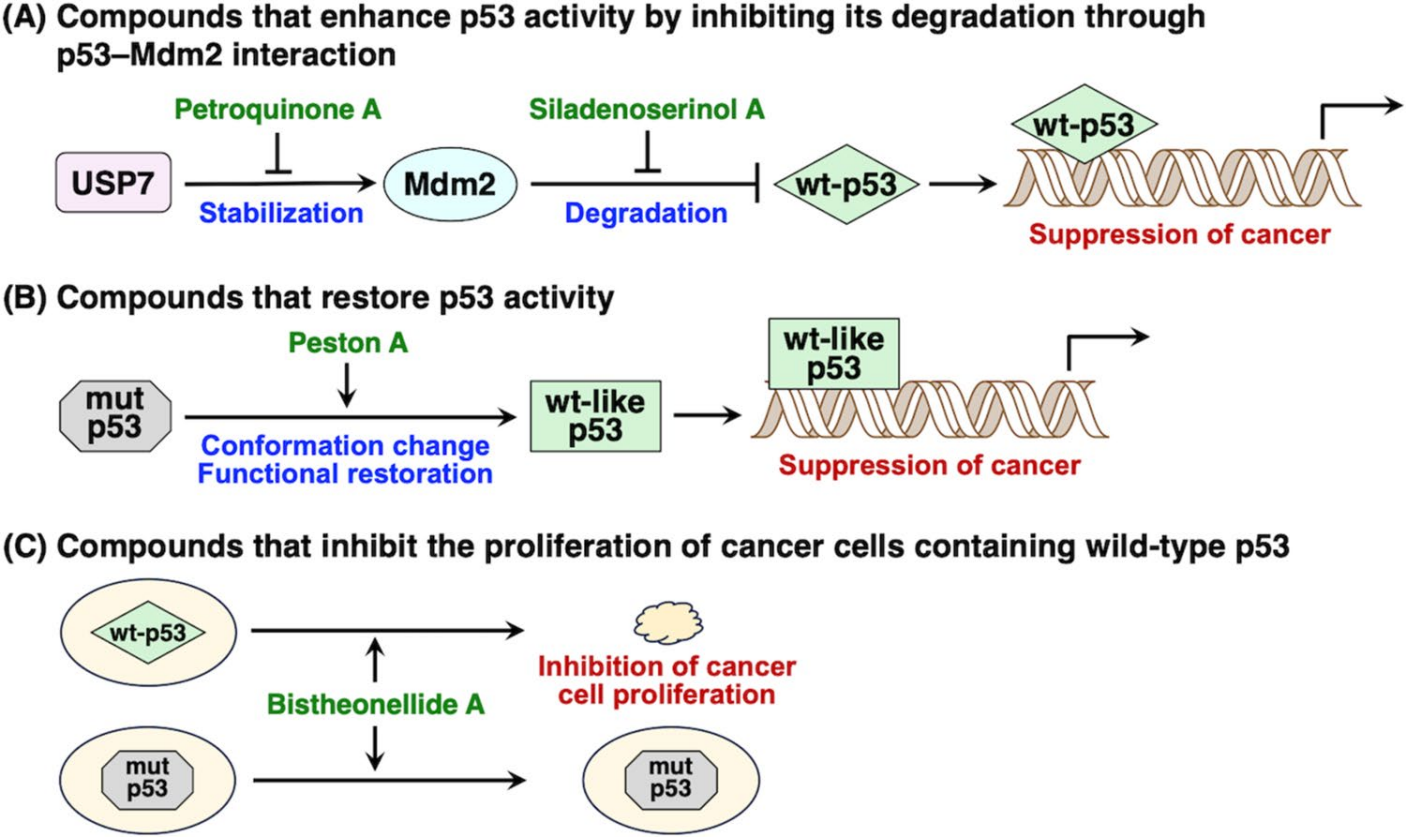

## Introduction

**Fig. 1:**
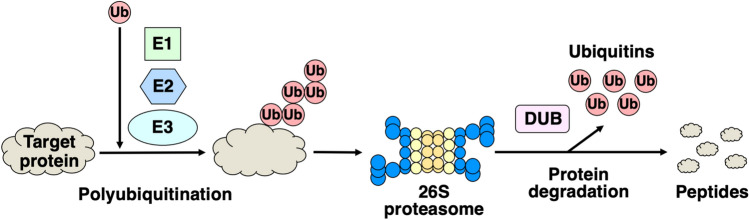
Protein degradation by the ubiquitin–proteasome system (UPS)

**Fig. 2:**
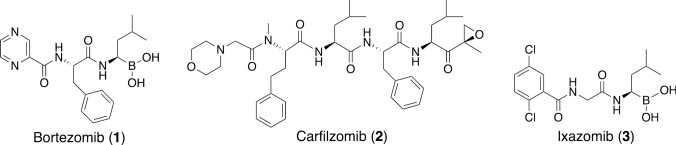
Structures of proteasome inhibitors approved by the FDA

Natural products have been particularly useful for drug discovery, and many FDA (U.S. Food and Drug Administration)-approved drugs contain natural products or their derivatives [1]. Recent studies have shown that, due to their low toxicity, natural products generally have a higher success rate in clinical trials than synthetic compounds [2]. Emerging technologies, such as cell-based high-throughput screening and machine learning approaches, have significantly contributed to the discovery of small molecule candidates from natural sources [3–6]. In our ongoing search for bioactive compounds from natural sources, we are interested in compounds that target the ubiquitin–proteasome system (UPS), which plays a major role in the selective degradation of proteins involved in the regulation of various cellular events (Fig. 1) [7–10]. The 26S proteasome degrades polyubiquitinated proteins through the sequential action of ubiquitin-activating enzyme (E1), ubiquitin-conjugating enzyme (E2), and ubiquitin ligase (E3). Polyubiquitin chains are removed by deubiquitinating enzymes (DUBs), and the protein moieties are degraded into peptides by the 26S proteasome. Several synthetic proteasome inhibitors, bortezomib (Velcade®) (**1**) [11], carfilzomib (Kyprolis®) (**2**) [12], and ixazomib (Ninlaro®) (**3**) [13] (Fig. 2), have been approved by the FDA for relapsed multiple myeloma, but drug development has primarily been focused on chemically modifying candidates that come from screening synthetic small molecule libraries. Because natural products have diverse structures and biological functions, the search for UPS inhibitors from natural sources may provide promising leads for drug development.

**Fig. 3:**
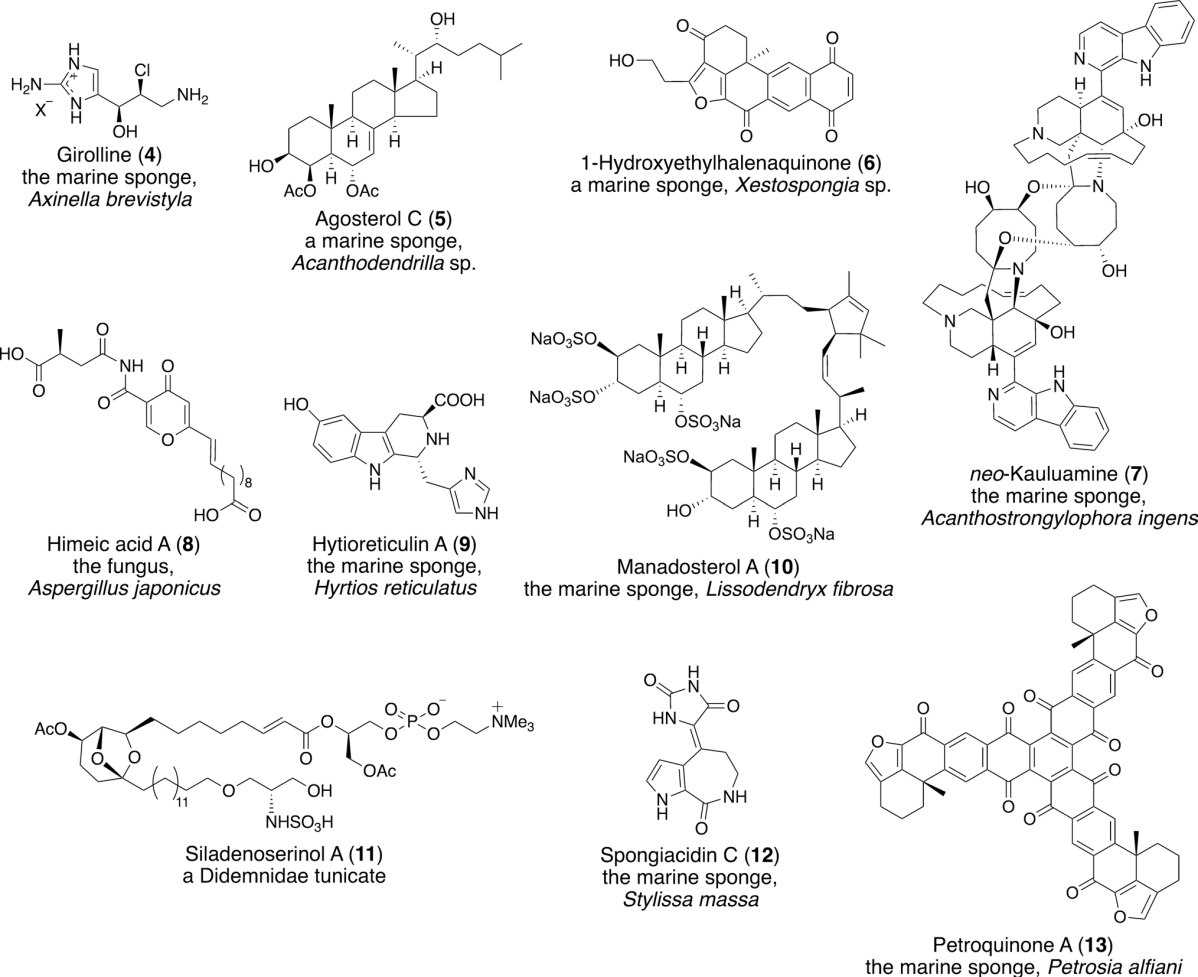
Structures and origins of UPS inhibitors isolated in our work

Our interest in the UPS began with the isolation of girolline (**4**) (Fig. 3) as a cytotoxic compound from the marine sponge, *Axinella brevistyla*, collected at Kamagi Bay on Sada Peninsula [14]. By studying its biological activity, we found that polyubiquitinated p53 was accumulated in HeLa cells upon treatment with girolline, and we speculated that girolline inhibits the recruitment of polyubiquitinated p53 to the proteasome [15]. This research led us to become interested in proteasome inhibitors. In 2003, we isolated our first proteasome inhibitors, agosterol derivatives (agosterol C (**5**)) (Fig. 3), from a marine sponge, *Acanthodendrilla* sp., collected on Noto Peninsula [16]. Agosterols were originally isolated from a marine sponge, *Spongia* sp., collected at Ago Bay by the Kobayashi group, and found to reverse multidrug resistance in tumor cells [17, 18], but our study was the first to show that agosterols exhibit proteasome inhibitory activity. After that, we investigated inhibitors of other components of the UPS, the ubiquitin system (E1, E2, and E3) and the deubiquitinating process mediated by DUBs (Fig. 1), as drug candidates, and we have been searching for UPS inhibitors from natural sources such as marine organisms and fungi. As a result, we isolated proteasome inhibitors (1-hydroxyethylhalenaquinone (**6**), IC_50_ 0.19 μM [19]; *neo*-kauluamine (**7**), IC_50_ 0.13 μM [20]), E1 inhibitors (himeic acid A (**8**), IC_50_ 50 μM [21]; hirtioreticulin A (**9**), IC_50_ 2.4 μM [22]), an inhibitor of the Ubc13–Uev1A interaction (manadosterol A (**10**), IC_50_ 0.090 μM [23]), an inhibitor of the p53–Mdm2 interaction (siladenoserinol A (**11**), IC_50_ 2.0 μM [24]), and USP7 inhibitors (spongiacidin C (**12**), IC_50_ 3.8 μM [25]; petroquinone A (**13**), IC_50_ 0.24 μM [26]) as representative compounds (Fig. 3).

Recently, in addition to inhibitors of p53 degradation that act through p53–Mdm2 interaction, we have become interested in p53 activators that function through various mechanisms. p53 is a tumor suppressor that induces the expression of various genes involved in DNA repair, cell cycle arrest, apoptosis, and senescence under cellular stress and DNA damage [27–29]. Therefore, enhancing p53 activity is a promising approach to suppress cancer [30], and we have been searching for compounds that target p53 from natural sources.

**Fig. 4:**
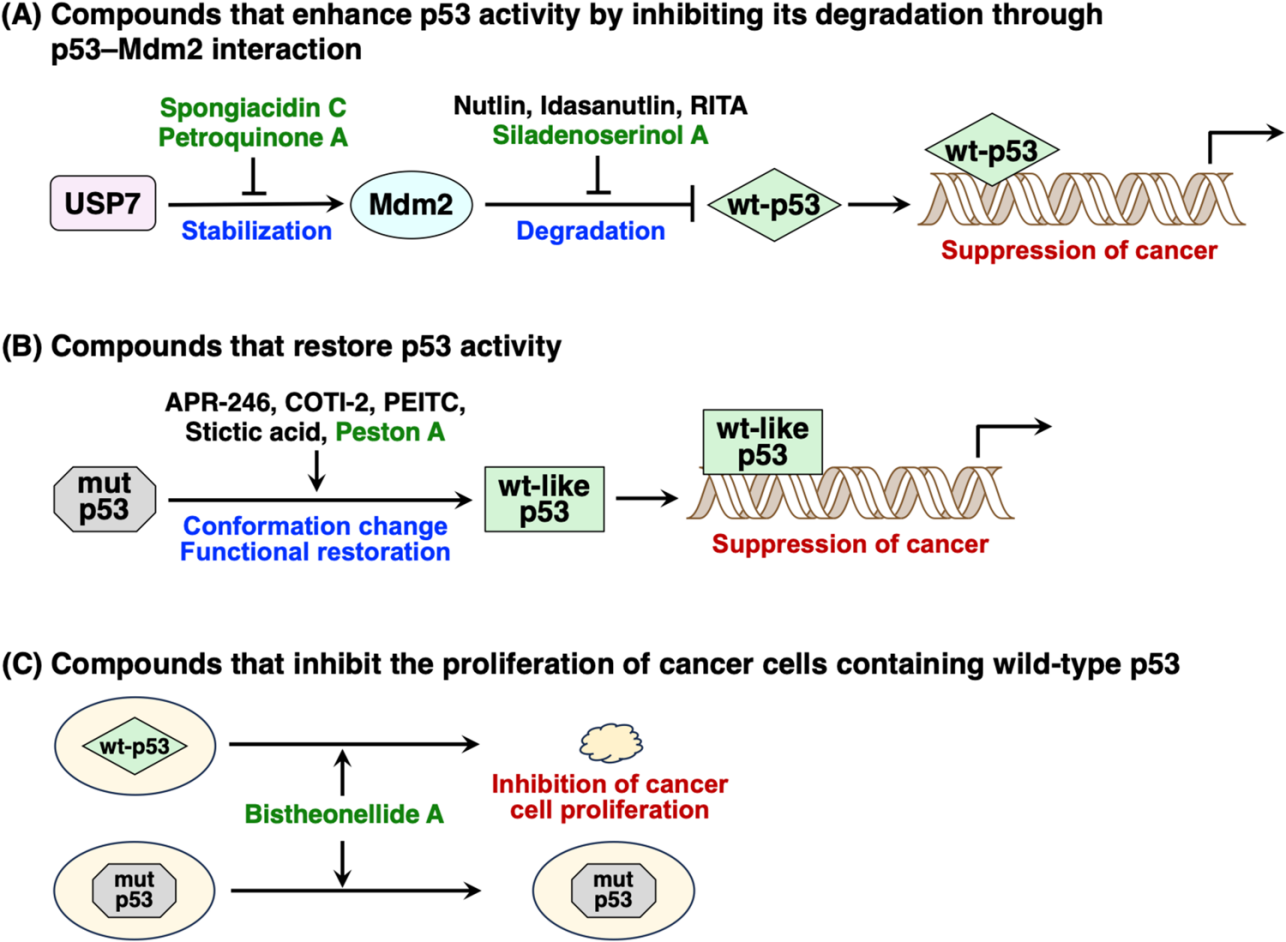
Three types of compounds that regulate p53 and suppress cancer cell proliferation. Compounds in green were isolated in our work. wt: wild-type, mut: mutant

This review summarizes natural products that regulate p53 and inhibit cancer proliferation. First, compounds that enhance p53 activity by inhibiting its degradation through p53–Mdm2 interaction are described (Fig. 4A). Next, considering that p53 is inactivated in more than 50% of human tumors, compounds that can reactivate mutant p53 through conformational changes and functional restoration are described (Fig. 4B). Finally, compounds that exhibit p53-dependent growth inhibition, excluding the inhibition of p53–Mdm2 interactions, are described, which are effective against tumors containing wild-type p53 (Fig. 4C).

## Natural products that enhance p53 activity by inhibiting its degradation through p53–Mdm2 interaction

**Fig. 5:**
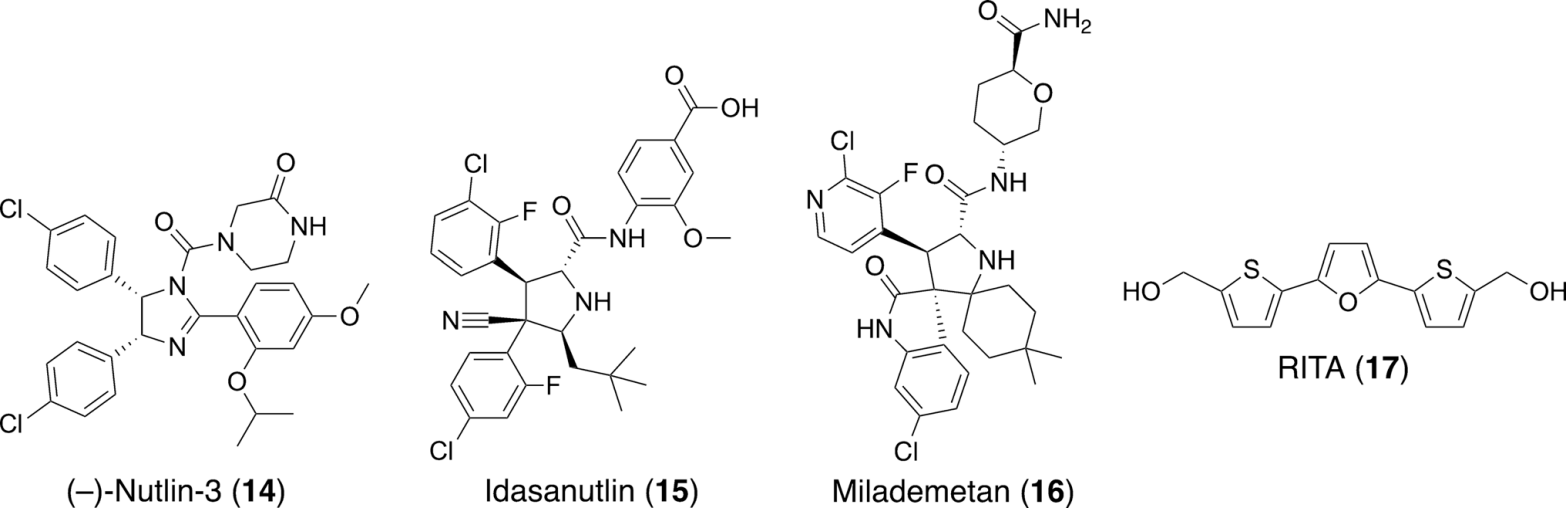
Structures of compounds that inhibit p53–Mdm2 interaction

Intracellular p53 levels are kept low and tightly regulated by the negative regulator Mdm2 (E3), which binds to p53 and induces its ubiquitynation and subsequent degradation by the proteasome [31, 32]. Mdm2 is normally expressed at low levels in cells but is overexpressed in cancer cells, thereby degrading large amounts of proteins. Hence, targeting Mdm2 is a promising approach to induce apoptosis and enhance p53 activity in cancer cells. In 2004, Vassilev and colleagues found that nutlins (nutlin-3 (**14**)) (Fig. 5) act as an Mdm2 antagonist, after searching a library of synthetic small molecules [33]. Nutlin-3 binds to the p53-binding pocket of Mdm2 and inhibits p53–Mdm2 binding, thus inhibiting p53 degradation and suppressing cancer growth [34]. Idasanutlin (**15**) (Fig. 5) is a nutlin derivative that binds to Mdm2 and prevents p53–Mdm2 interaction [35], and a phase III clinical trial of idasanutlin in combination with cytarabine in patients with relapsed or refractory acute myeloid leukemia was conducted. Milademetan (**16**) (Fig. 5) is an Mdm2 inhibitor that has demonstrated therapeutic efficacy in phase Ib/II studies in patients with intimal sarcoma, an extremely rare cancer with no standard care [36, 37]. A phase III study is underway to evaluate the safety and efficacy of milademetan compared with trabectedin in patients with dedifferentiated liposarcoma [38]. Meanwhile, a small molecule, RITA (reactivation of p53 and induction of tumor cell apoptosis) (**17**) (Fig. 5), binds to p53 and inhibits p53–Mdm2 interaction, showing a significant p53-dependent antitumor effect in vivo [39]. These data indicate that p53–Mdm2 inhibitors may lead to the development of anticancer drugs targeting tumors with wild-type p53. Then, we have been screening extracts of natural sources to find structurally unique p53–Mdm2 inhibitors. Extracts of fungi and marine invertebrates were screened against the inhibition of p53–Mdm2 binding by ELISA with purified recombinant p53 and Mdm2 proteins and the primary anti-Mdm2 antibody.

**Fig. 6:**
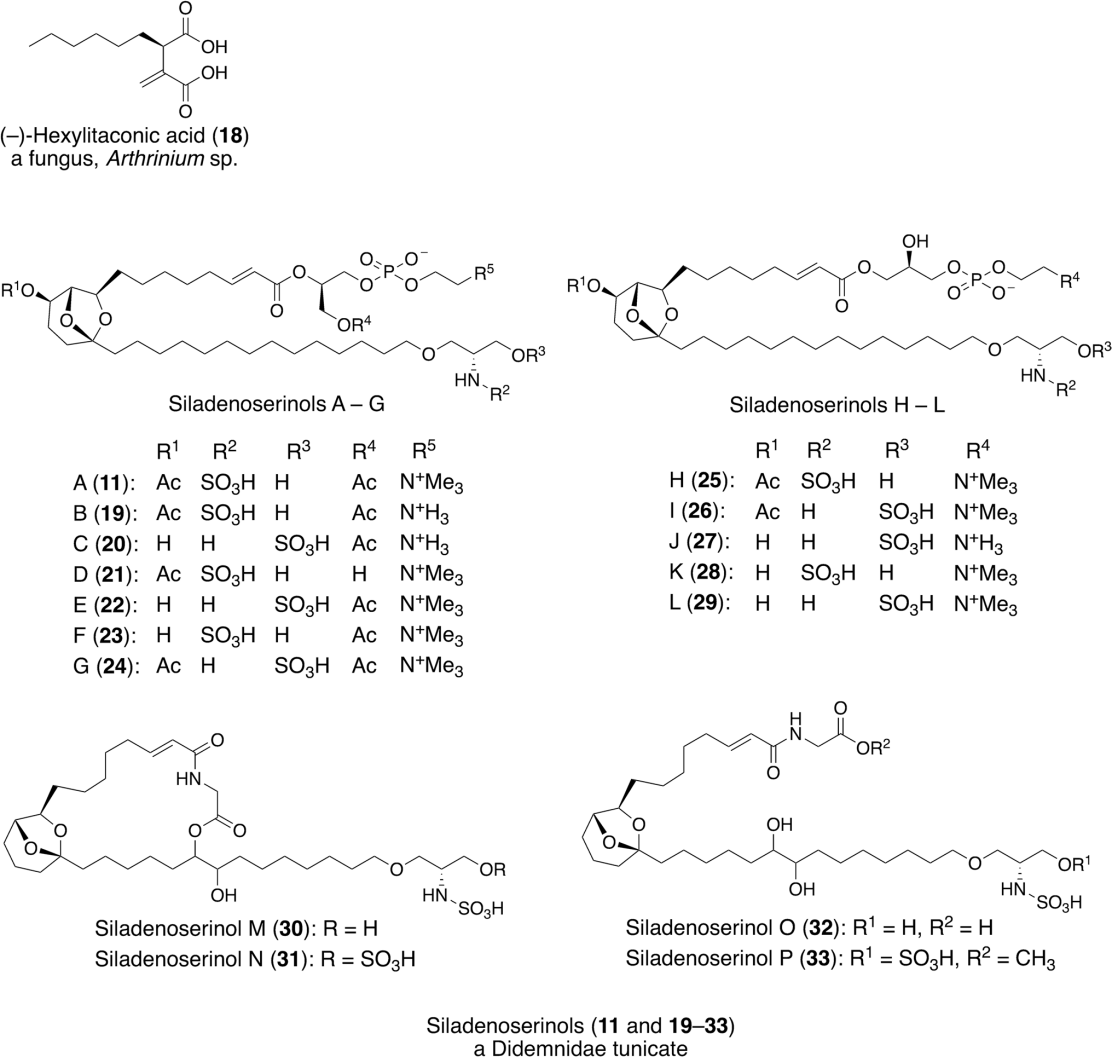
Structures and origins of compounds that inhibit p53–Mdm2 interaction, isolated in our work

We isolated (–)-hexylitaconic acid (**18**) (Fig. 6) as an inhibitor of p53–Mdm2 interaction from an extract of a fungus, *Arthrinium* sp., which was separated from an unidentified marine sponge collected on Noto Peninsula, with an IC_50_ value of 50 μg/mL [40, 41]. Although many synthetic inhibitors of the p53–Mdm2 interaction had been previously reported, (–)-hexylitaconic acid was the second naturally derived inhibitor. Subsequently, we isolated siladenoserinols A–P (**11** and **19**–**33**, respectively) (Fig. 6) [24, 42] from a tunicate of the family Didemnidae collected in Indonesia. Siladenoserinols were determined to be novel sulfonated serinolipids containing a 6,8-dioxabicyclo[3.2.1]octane unit. While siladenoserinols A–L have either a glycerophosphocholine or glycerophosphoethanolamine moiety, siladenoserinols M–P lack them. Siladenoserinols M and N have macrocyclic structures with additional glycine units connected by amide and ester bonds, and the ester bonds are cleaved in siladenoserinols O and P. Siladenoserinols A–L inhibited the p53–Hdm2 interaction with IC_50_ values ranging from 2.0 to 55 μM. Siladenoserinols A and B were the most potent inhibitors, both with an IC_50_ value of 2.0 μM, whereas siladenoserinols M–P showed no inhibition, even at 50 μM. These results indicate that the acetyl or hydroxyl groups on the bicyclic ketal moiety and the glycerophospholipid moiety are important for the inhibition of p53–Hdm2 interaction.

Among the inhibitors of UPS components, E3 inhibitors are expected to be more effective and less toxic than proteasome inhibitors because each E3 specifically recognizes a target protein. Furthermore, DUB inhibitors are expected to be specific drug candidates. The human genome is reported to encode at least 98 DUBs, and changes in DUB function are associated with multiple diseases, including cancer [43–45]. Among DUBs, USP7 stabilizes Mdm2 and represents an emerging target for cancer therapy [46, 47]. Mdm2 is self-ubiquitynated and subsequently degraded by the proteasome, whereas USP7 removes ubiquitin from self-ubiquitynated Mdm2, leading to stabilization of Mdm2 and subsequent degradation of p53. Therefore, USP7 inhibitors stabilize intracellular p53 by degrading Mdm2, which leads to cancer suppression. To date, USP7 inhibitors have been developed by screening synthetic small molecule libraries and chemically modifying lead compounds. As an alternative approach, we screened a library of natural source extracts and isolated inhibitors from the hits.

**Fig. 7:**
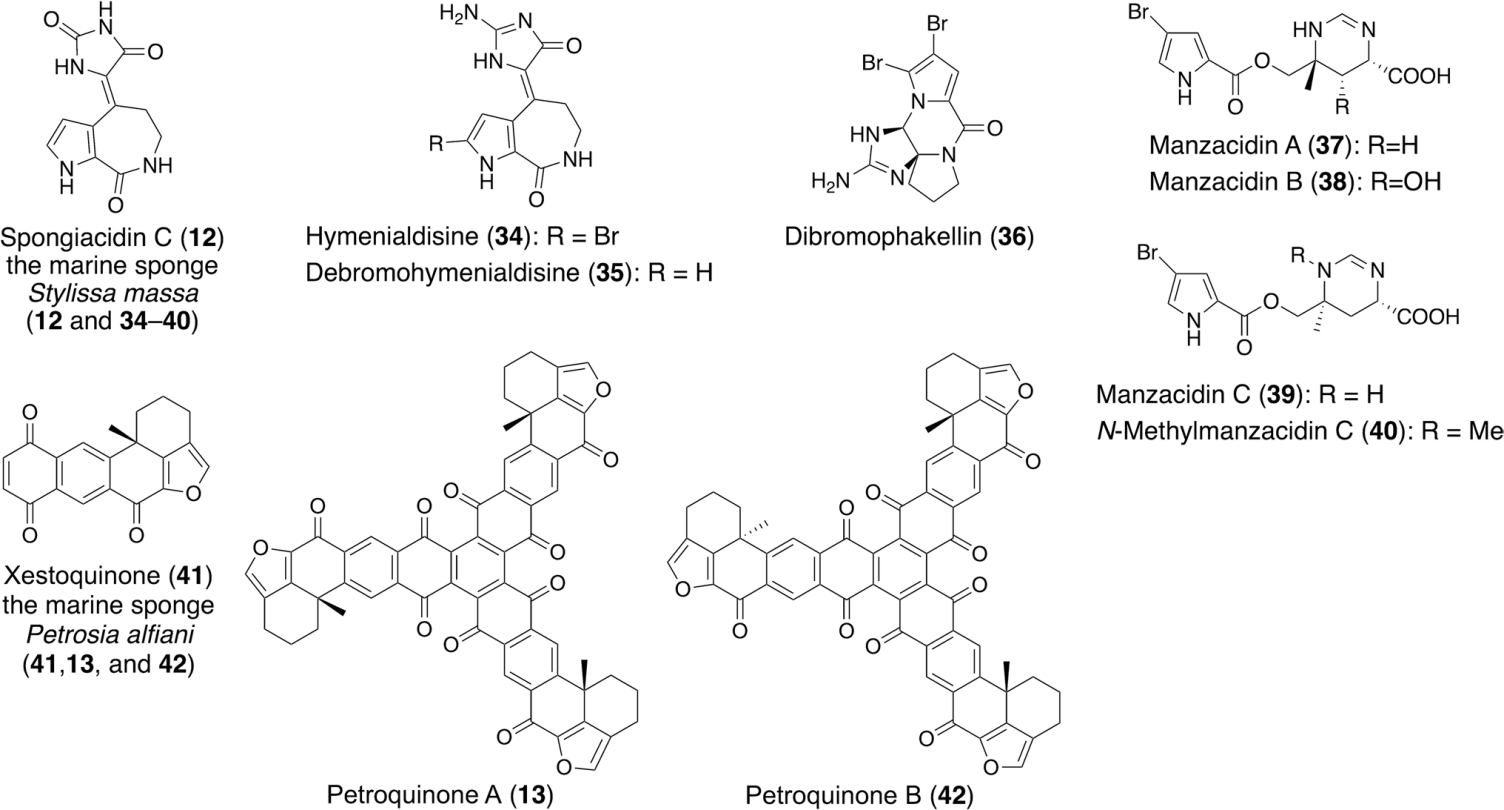
Structures and origins of compounds that inhibit USP7, isolated in our work

We isolated spongiacidin C (**12**), hymenialdisine (**34**), debromohymenialdisine (**35**), dibromophakellin (**36**), manzacidin A (**37**), manzacidin B (**38**), manzacidin C (**39**), and *N*-methylmanzacidin C (**40**) from the marine sponge, *Stylissa massa*, collected in Indonesia (Fig. 7) [25]. Among these compounds, spongiacidin C was the strongest USP7 inhibitor, with an IC_50_ value of 3.8 μM. Hymenialdisine and debromohymenialdisine showed 20% inhibition at 20 μM, and the other alkaloids were inactive. These results indicated that the presence of the hydantoin moiety in spongiacidin C increases the USP7 inhibitory activity compared with the aminoimidazolinone moiety in hymenialdisine and debromohymenialdisine. Notably, the inhibitory effect of spongiacidin C was stronger against USP7 than USP21, USP2, USP8, or SENP1, and therefore, the effect may be considered specific to USP7. Although several natural products with broad DUB inhibitory profiles have been isolated [48], spongiacidin C is the first natural USP7 inhibitor and exhibits potency comparable to synthetic USP7 inhibitors.

In addition, we isolated 16 new xestoquinone derivatives (two trimers, six dimers, and four monomers) from the marine sponge, *Petrosia alfiani*, collected in Indonesia (Fig. 7) [26]. The strongest USP7 inhibitor is xestoquinone (**41**) (IC_50_, 0.13 μM), but petroquinones A (**13**) and B (**42**) (IC_50_, 0.75 and 0.36 μM, respectively) also showed strong inhibition. USP7 belongs to the cysteine protease family, and the presence of α,β-unsaturated carbonyl moieties in the compounds may be involved in the inhibition mechanism.

## Natural products that restore p53 activity

In over 50% of tumor cells, p53 is inactivated by mutations [27, 49, 50], and mutant p53 aggregates within cells to promote cancer progression, invasion, and metastasis [51, 52]. Such mutations may inhibit direct binding to DNA or cause structural perturbations that prevent the tumor suppressor from folding properly or forming oligomers. Therefore, compounds that change mutant p53 to wild-type-like p53 may lead to cancer suppression, and several compounds have been reported to restore p53 activity (Fig. 4B) [29, 49, 53].

**Fig. 8:**
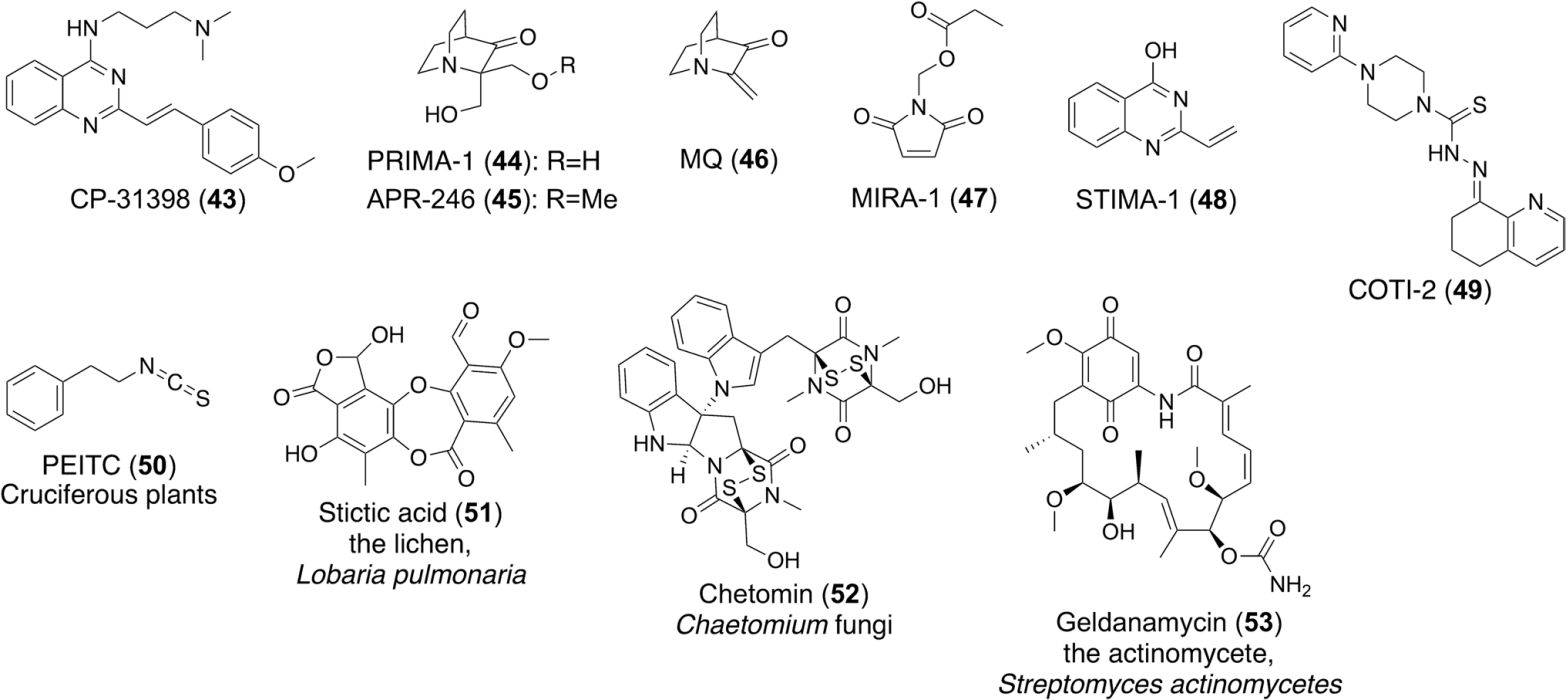
Structures and origins of compounds that activate mutant p53

In 1999, the Foster group searched a synthetic compound library for compounds that allowed mutant p53 to retain an active conformation and discovered CP-31398 (**43**) (Fig. 8), which restored transcriptional activity of the p21 gene in cells expressing mutant p53 and inhibited tumor growth in mice [54].

In 2002, the Bykov group discovered PRIMA-1 (**44**) (Fig. 8) from a compound library, which restores the transcriptional transactivation function to mutant p53 in cells expressing mutant p53 [55]. They compared the activity of several PRIMA-1 analogs and found that its methyl derivative, APR-246 (PRIMA-1^MET^) (**45**) (Fig. 8), was more active than PRIMA-1 [56]. PRIMA-1 and APR-246 are hydrolyzed in vivo and converted to methylene quinuclidinone (MQ) (**46**) (Fig. 8) to exert antitumor activity [57]. MQ forms adducts with thiols in mutant p53, and covalent modification of mutant p53 induces apoptosis in tumor cells, demonstrating the concept of more potent and specific anticancer drugs that target mutant p53. Computational methods revealed that the binding site of PRIMA-1 is the Cys124 residue, located in the center of the pocket between loop L1 and sheet S3 of the p53 core domain [58]. APR-246 showed a synergized chemotherapeutic effect with the combination of clinically used drugs. Currently, a phase III trial is underway in combination with 5-azacytidine in patients with *TP53*-mutated myelodysplastic syndromes (MDS), a type of blood cancer [59]. In addition, the Mykov group reported that MIRA-1 (**47**) (Fig. 8) [60], a maleimide derivative, and STIMA-1 (**48**) (Fig. 8) [61], a simple derivative of CP-31398, reactivated DNA binding of mutant p53, restored transcriptional activity of mutant p53 in cells, and induced mutant p53-dependent cell death in human tumor cell lines. They were also shown to bind to the Cys124 residue within the L1/S3 pocket of mutant p53 [58].

COTI-2 (**49**) (Fig. 8) is an orally available thiosemicarbazone that binds to the misfolded mutant p53 protein, induces a conformational change, and restores its activity. It is currently undergoing a phase I clinical trial in patients with advanced or recurrent gynecologic and head and neck malignancies [62].

In addition to the synthetic compounds described above, several natural products that reactivate mutant p53 have been reported. Phenethyl isothiocyanate (PEITC) (**50**) (Fig. 8) [63] was isolated from Cruciferous plants and reactivated mutant p53 under in vitro and in vivo conditions. PEITC sensitized the p53^R175H^ mutant to degradation by the proteasome and autophagy and inhibited tumor growth in the xenograft mouse model through dietary supplementation. This result may be beneficial for primary health care and primary cancer prevention. The effect of watercress supplementation on cancer progression and recurrence was examined in a phase III clinical trial through nutritional supplementation in cancer patients [38].

Virtual screening identified stictic acid (**51**) (Fig. 8) as a potential p53-reactivating compound [58]. Stictic acid was originally isolated from the lichen *Lobaria pulmonaria* and was more potent than PRIMA-1 in reactivating p21 expression in human osteosarcoma cells with mutant p53^R175H^, suggesting that the L1/S3 pocket is a target for pharmacological activation of p53 mutant.

Moreover, several compounds have been reported to enhance p53 function through the involvement of chaperone molecules. Chetomin (**52**) (Fig. 8) is produced by fungi of the genus *Chaetomium* and increases the binding of Hsp40 to the p53^R175H^ mutant, which induces a conformational change to wild-type-like p53, thereby restoring the transcriptional activity of p53 [64]. In addition, mutant p53 evades Mdm2-mediated degradation by binding to Hsp90, but when geldanamycin (**53**) (Fig. 8), which is produced by *Streptomyces actinomycetes*, binds to Hsp90, mutant p53 is liberated from the complex and subsequently degraded by Mdm2 and CHIP, suppressing cancer progression [65].

### Colletofragarone A2 promotes the degradation and aggregation of mutant p53, suppressing cancer cell proliferation in vivo

**Fig. 9:**
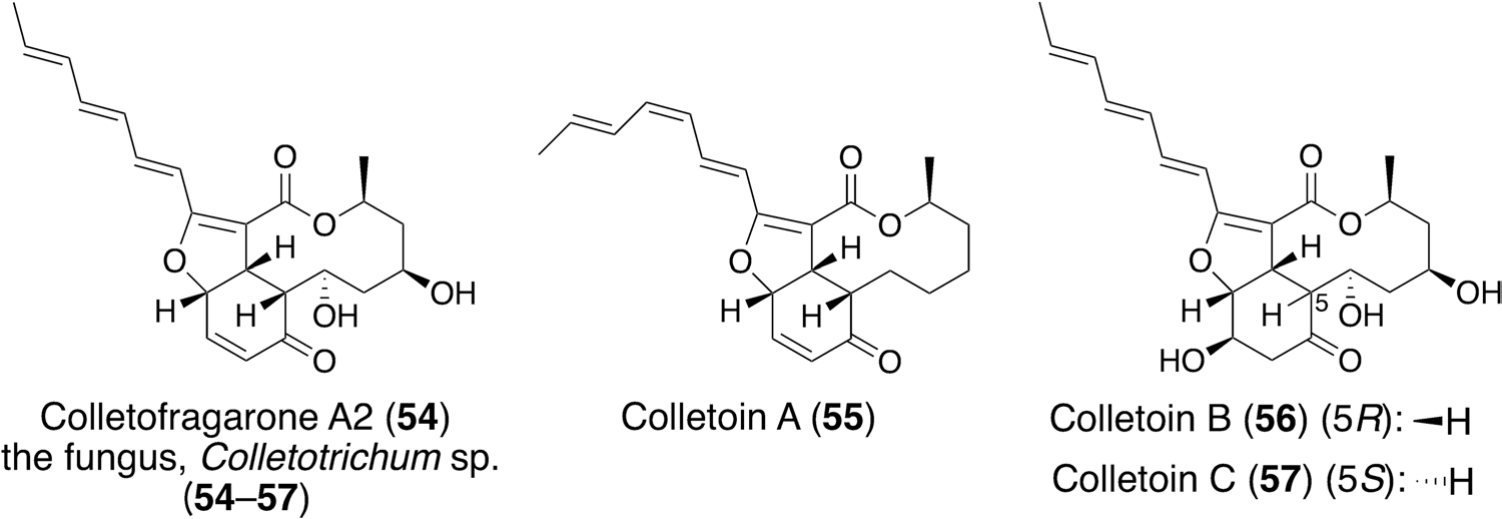
Structures and an origin of colletofragarone A2 and colletoin A–C, isolated in our work

As described above, various compounds that reactivate mutant p53 have been reported, and their mechanisms were investigated. However, most of them are synthetic compounds, and we searched for mutant p53-reactivators from natural sources. First, we examined cell viability, because compounds that reduce mutant p53 levels are expected to suppress tumor growth. Among 2311 fungal extracts, 166 samples showed cytotoxicity and were therefore subjected to immunofluorescence staining with two anti-p53 antibodies, namely DO-1 and Pab240 antibodies, using Saos-2 (p53^R175H^) cells. The DO-1 antibody detected both wild-type(-like) and mutant p53, whereas the Pab240 antibody recognized only mutant p53. From the seven samples selected by immunofluorescence staining, we investigated active compounds of the fungus, *Colletotrichum* sp., isolated from a plant in Kumamoto Prefecture. Bioassay-guided purification of the fungal extract cultured on rice medium (1 kg) yielded colletofragarone A2 (**54**) (9.3 mg) and three new derivatives, colletoins A (**55**) (23.9 mg), B (**56**) (4.3 mg), and C (**57**) (1.8 mg) (Fig. 9) [66].

Colletofragarone A2 was originally isolated as a germination self-inhibitor from *C. fragariae* by the Ueno group [67]. Although the relative configurations of three carbons in the 10-membered lactone were not reported in the literature, we determined the relative and absolute configurations of all carbons by spectroscopy and DFT calculations. Among the four compounds, colletofragarone A2 and colletoin A were more cytotoxic (IC_50_, 0.35 and 0.36 μM, respectively) than PEITC (1.3 μM) in Saos-2 (p53^R175H^) cells. Notably, colletoins B and C were less potent (21 and 12 μM, respectively), indicating that the α,β-unsaturated carbonyl group is involved in the cytotoxicity. Next, the antitumor effect of colletofragarone A2 was tested in a xenograft mouse model. HuCCT1 cells expressing p53^R175H^ were subcutaneously injected into nude mice, and colletofragarone A2 was intratumorally injected every other day for 13 days. HuCCT1 cells were more firmly fixed in mice than Saos-2 (p53^R175H^) cells and were used for in vivo testing, showing that colletofragarone A2 reduced tumor volumes without weight loss [68].

To examine whether colletofragarone A2 restored p53 function, colletofragarone A2-treated Saos-2 (p53^R175H^) cells were analyzed by immunofluorescence staining with DO-1 and Pab240 antibodies. Intracellular fluorescence levels were reduced by staining with the Pab240 antibody but were unchanged with the DO-1 antibody, showing that colletofragarone A2 can change the structure of mutant p53 to wild-type-like p53 and restore its function. To confirm this, the intracellular p53 level was analyzed by western blotting. Then, colletofragarone A2-treated SK-BR-3 (p53^R175H^) cells were extracted by NP-40 buffer, and the solution was analyzed by western blotting with the DO-1 antibody. The results showed that colletofragarone A2 reduced p53 levels in a dose-dependent manner, which was not consistent with the immunofluorescence staining results. This indicated that the insoluble fraction in NP-40 buffer likely contains p53. To completely extract the protein, colletofragarone A2-treated cells were extracted with SDS buffer at 95 °C. p53 slightly decreased in a dose-dependent manner in the SDS buffer lysate, but more p53 was detected in the SDS buffer lysate than in the NP-40 buffer lysate. These results suggest that colletofragarone A2 induced the aggregation of mutant p53 [68], similar to molecular chaperon inhibitors such as geldanamycin [69] and gambogic acid [70].

Ultimately, colletofragarone A2 reduced intracellular mutant p53 levels and inhibited the proliferation of mutant p53-expressing cells in vivo. To examine whether the reduction in the intracellular level of mutant p53^R175H^ was caused by proteolysis, we investigated the effect of colletofragarone A2 on p53 levels in SK-BR-3 (p53^R175H^) cells in the presence of a protein synthesis inhibitor, cycloheximide. In this experiment, colletofragarone A2 promoted the reduction in p53 levels, suggesting that colletofragarone A2 reduced the levels by promoting the degradation of mutant p53^R175H^ protein in cells. To confirm this, we examined the involvement of the proteasome in the degradation of mutant p53 in SK-BR-3 cells. Cells were treated with colletofragarone A2, with or without the proteasome inhibitor, MG132, and extracted with 1% NP-40 buffer. Insoluble precipitates and aggregates of mutant p53 in NP-40 buffer were dissolved in 2% SDS buffer at 95 °C, and the solution was analyzed by western blotting. In the presence of MG132, colletofragarone A2 slightly decreased mutant p53 levels in the NP-40 buffer-soluble fraction, but increased levels in the insoluble fraction. The results indicate that colletofragarone A2 promoted proteasome-mediated degradation of mutant p53 and the accumulation of aggregated mutant p53 [68].

In summary, mutant p53 is unstable alone but has been stabilized by binding to chaperones. Colletofragarone A2 may act on molecular chaperones to release mutant p53 from the complex, and the released unstable mutant p53 is degraded by the proteasome and aggregated.

### Pestone A binds to mutant p53 and changes its conformation, suppressing cancer cell proliferation in vivo

**Fig. 10:**
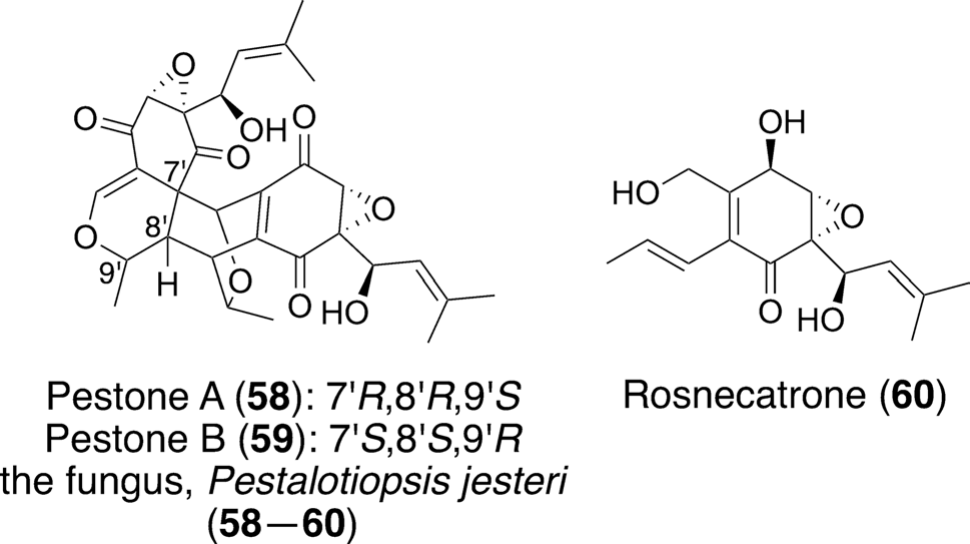
Structures and an origin of pestones A and B and rosnecatrone, isolated in our work

To find other reactivators of mutant p53, 7701 extracts prepared from fungi and marine organisms were screened for cell viability, and 970 hits were examined by immunofluorescence staining using DO-1 and Pab240 antibodies in Saos-2 (p53^R175H^) cells. Among 15 samples with reduced mutant p53 levels, the fungus *Pestalotiopsis jesteri*, isolated from legume leaves in Miyazaki Prefecture, was selected for further study. The fungus was grown in rice medium (1.4 kg) and extracted with *n*-BuOH. Bioassay-guided purification of the extract afforded new compounds, pestones A (**58**) (20.6 mg) and B (**59**) (5.1 mg) [71], and a known compound, rosnecatrone (**60**) (2.05 g) (Fig. 10) [72]. Spectroscopic analysis showed that pestones A and B have the same planar structure with dimeric features derived from rosnecatrone. Analysis of the NOE correlations showed that their configurations differed at C-7', C-8', and C-9'. From a biogenetic perspective, pestones A and B may have been formed from rosnecatrone by the cascade reaction involving oxidation, 6π-electrocyclization, and the Diels–Alder reaction [71]. To confirm the absolute configurations, pestones A and B were semi-synthesized from rosnecatrone, and the ^1^H NMR and ECD spectra of synthetic pestones A and B were identical to those of natural products. Thus, the absolute configurations of pestones A and B were determined.

Saos-2 (p53^R175H^) cells were treated with pestones A (2 μM) or B (2 μM) and analyzed by immunofluorescence staining using Pab240 and DO-1 antibodies. Treatment with pestones A or B did not change the fluorescence level detected by the DO-1 antibody but did change the level detected by the Pab240 antibody. This indicated that pestones A and B both reduced the mutant p53 level. We next examined the binding of pestone A to mutant p53 in a cellular thermal shift assay using Saos-2 (p53^R175H^) cells. Immunoblot analysis showed that the treatment of cells with 5 μM of pestone A induced the thermal stability of mutant p53 at 40 and 43 °C, revealing that the compound binds to mutant p53 in Saos-2 (p53^R175H^) cells [71].

Pestones A and B were cytotoxic, with IC_50_ values of 1.0 and 1.1 μM, respectively, whereas the IC_50_ value of rosnecatrone was over 50 μM. This suggests that the unsaturated carbonyl groups of pestones A and B are responsible for their cytotoxicity. Considering that p53 was reported to induce apoptosis, we performed flow cytometry analysis by employing an Annexin V-FITC/PI staining assay using Saos-2 (p53^R175H^) cells. Treatment of the cells with pestone A (4 μM) increased the early and late apoptotic cells compared with the control, indicating that pestone A induced apoptosis. Overall, the biological activities shown by pestone A suggest an antitumor effect. Intratumoral injection of pestone A into tumor-bearing mice reduced tumor volume without weight loss [71].

Pestone A likely binds to mutant p53 and alters its conformation, but the details of the mechanism remain unclear.

## Natural products that inhibit the proliferation of cancer cells containing wild-type p53

Compounds that inhibit p53–Mdm2 binding release p53 and increase p53 activity in cells. Moreover, Mdm2 is amplified and overexpressed in cancer cells, and compounds that promote Mdm2 degradation in cells or inhibit Mdm2 activity are also powerful drug candidates for cancers that harbor wild-type p53. The Weissman group reported a series of small molecules (HLI98s) that inhibit human Mdm2 activity [73]. Therefore, we searched for compounds from natural sources that exhibit p53-dependent growth inhibitory activity, which may inhibit the proliferation of cancer cells containing wild-type p53.

**Fig. 11:**
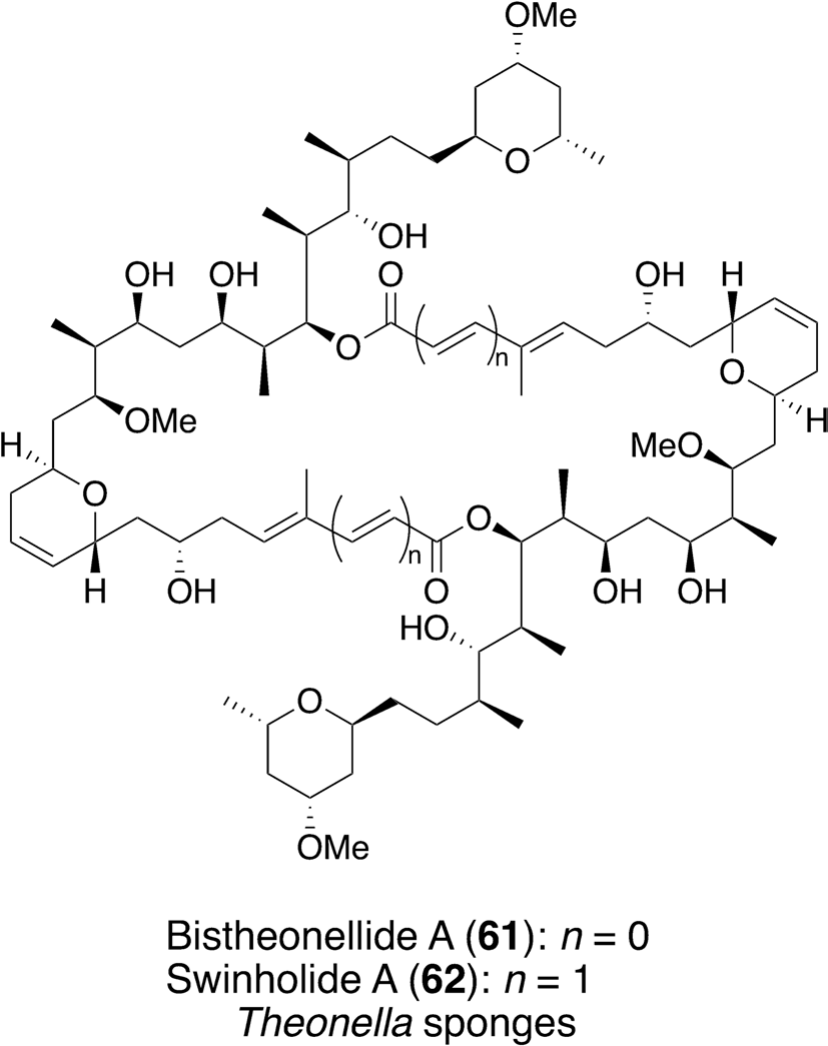
Structures and an origin of bistheonellide A and swinholide A

Screening was performed using two colorectal cancer cell lines: wild-type p53-containing HCT116 (p53^+/+^) cells and p53-null HCT116 (p53^–/–^) cells [74]. Among 1232 marine sponge extracts, an extract of the *Theonella* sponge collected in Indonesia revealed over 30% differentiation for the cell proliferation between these two cell lines in a concentration-dependent manner. Analysis of the extract using bioactivity-based molecular networking [75] suggested that bistheonellide A (**61**) (Fig. 11) was the active compound, which was obtained by conducting bioassay-guided purification of the sponge extract. The IC_50_ values of bistheonellide A were 2.7 and 33 nM againt HCT116 (p53^+/+^) and HCT116 (p53^–/–^) cells, respectively, and it was 12-fold more toxic to HCT116 (p53^+/+^) cells than to HCT116 (p53^–/–^) cells. To examine whether this effect was p53-dependent, p53-regulated transcriptional activation of p21 was analyzed. Western blotting analysis of the lysate from bistheonellide A-treated HCT116 (p53^+/+^) cells showed a concentration-dependent increase in p21 expression, whereas no change was observed in HCT116 (p53^–/–^) cells. This result indicates that bistheonellide A exhibited p53-dependent activity [76]. Bistheonellide A was originally isolated from the *Theonella* marine sponge collected by the Fusetani group on Hachijo island and inhibited the development of starfish embryos [77]. Although the target molecule is unknown, this is the first report showing that bistheonellide A exhibits a specific growth inhibitory effect on cells containing wild-type p53.

Swinholide A (**62**) (Fig. 11) is structurally similar to bistheonellide A and was originally isolated as a cytotoxic compound from the marine sponge, *Theonella swinhoei*, collected in Okinawa [78, 79]. Unlike bistheonellide A, swinholide A exhibited similar cytotoxicities against both HCT116 (p53^+/+^) and HCT116 (p53^–/–^) cells, with IC_50_ values of 6.1 and 6.3 nM, respectively. Swinholide A is a 44-membered ring macrodiolide that contains an additional methylene unit next to each carbonyl carbon compared with bistheonellide A, indicating that the difference in ring size is associated with the p53-specific toxicity of bistheonellide A [76].

## Conclusion

This review summarized the structures and functions of compounds targeting wild-type and mutant p53 (Fig. 4). Cancer treatments that target p53 leverage the cancer-suppressing effect of p53, which is inherent in the body, and are therefore expected to have few side effects. In addition, when multiple drugs that act on different targets are used in combination to enhance the action of p53, synergistic effects may significantly improve the therapeutic efficacy. Cancer therapeutics based on the tumor-suppressing effect of p53 have the potential to become a new pharmaceutical model.
